# Atypical mast cells and multiple myeloma: An ambiguous alliance with diagnostic implications

**DOI:** 10.1111/bjh.70352

**Published:** 2026-02-15

**Authors:** Jean‐Baptiste Rieu, Anaïs Schavgoulidze, Alban Canali, Laetitia Largeaud, Aurore Perrot, Charlotte Syrykh

**Affiliations:** ^1^ Laboratoire d'Hématologie, Centre Hospitalo‐Universitaire (CHU) de Toulouse Institut Universitaire du Cancer de Toulouse‐Oncopole (IUCT‐O), Université de Toulouse, UPS Toulouse France; ^2^ Service d'Hématologie, Centre Hospitalo‐Universitaire (CHU) de Toulouse Institut Universitaire du Cancer de Toulouse‐Oncopole (IUCT‐O), Université de Toulouse, UPS Toulouse France; ^3^ Laboratoire d'Anatomopathologie, Centre Hospitalo‐Universitaire (CHU) de Toulouse Institut Universitaire du Cancer de Toulouse‐Oncopole (IUCT‐O), Université de Toulouse, UPS Toulouse France

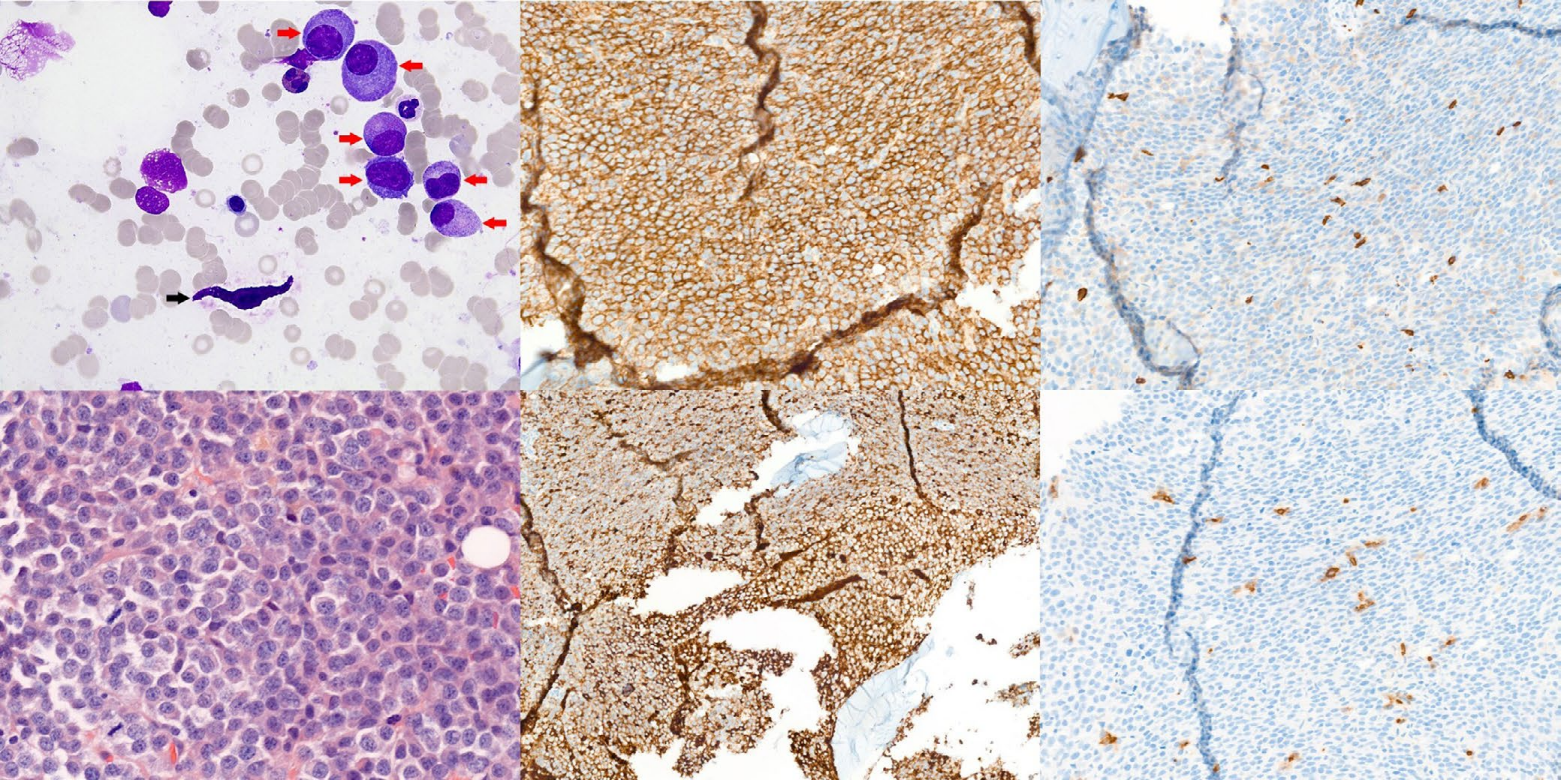

An 84‐year‐old man presented with anaemia (Hb 70 g/L), monoclonal Immunoglobulin A (IgA) kappa spike (13.6 g/L) and elevated kappa free light chains (216 mg/L, kappa/lambda ratio 4.9). White blood cell and platelet counts were normal, without eosinophilia. Serum calcium and creatinine were normal. Skeletal imaging showed no osteolytic lesions. Physical examination revealed no hepatosplenomegaly or cutaneous lesions. The patient reported a self‐limited episode of diarrhoea but no history of flushing or anaphylaxis. Bone marrow (BM) aspirate showed 35% dysmorphic plasma cells (PCs) (top left image; May–Grünwald–Giemsa staining, ×63 objective; red arrows), consistent with multiple myeloma (MM), along with approximately 1% spindle‐shaped mast cells (MCs) (top left image; black arrow). Cytogenetic analysis of PCs showed a 44‐chromosome hypodiploid karyotype with monosomies of 13 and 22, gains of 1q and 20q and loss of 20p. BM trephine biopsy (lower left image; haematoxylin and eosin staining, ×40 objective) confirmed infiltration by neoplastic CD138+ (top middle image; immunostaining, ×20 objective) kappa+ (lower middle image; immunostaining, ×10 objective) PCs, associated with dispersed CD117+ (top right image; immunostaining, ×20 objective) and tryptase+ (lower right image; immunostaining, ×20 objective) MCs, 30% of which were spindle‐shaped, expressing aberrant CD2/CD25 by flow cytometry but lacking dense aggregates. Serum tryptase was normal (3.57 μg/L) and no *KIT* mutation was detected by next‐generation sequencing (exons 8, 9, 11, 13 and 17) and allele‐specific quantitative polymerase chain reaction (PCR) targeting D816V (limit of detection 0.01% variant allele frequency). These findings support MM with reactive MC hyperplasia rather than systemic mastocytosis with an associated haematological neoplasm (SM‐AHN).

MCs are increasingly recognised as important contributors to MM pathogenesis, promoting PC growth, angiogenesis and stromal remodelling through cytokine release and stromal interactions.^1^, ^2^ Yet their atypical morphology may also suggest SM‐AHN, a rare entity more often associated with myeloid neoplasms but also reported with MM.^3^ This case underscores the diagnostic ambiguity caused by atypical MC morphology in MM and highlights the need for comprehensive morphologic, immunophenotypic and molecular evaluation to prevent diagnostic pitfalls.

## FUNDING INFORMATION

Centre Hospitalier Universitaire de Toulouse.

## PATIENT CONSENT STATEMENT

This manuscript respects ethic policy of CHU Toulouse for treatment of human research participants. The authors did not obtain written informed consent from the patient but the patient did not object to his data being used for research purposes (as required by ethic policy of CHU Toulouse). Written permission for reproduction from the copyright owners will be provided if the submission is accepted.
